# O3-7 Prenatal predictors and physical fitness in Spanish Youth: the UP&DOWN study

**DOI:** 10.1093/eurpub/ckac094.023

**Published:** 2022-08-29

**Authors:** Carmen Padilla-Moledo, Jose Castro-Piñero, A Perez-Bey, J Conde-Caveda, Irene Esteban-Cornejo, Oscar L Veiga, K. D Hesketh, Narcis Gusi

**Affiliations:** 1 Department of Physical Education, Faculty of Education Sciences, University of Cadiz, Puerto Real, Spain; 2 Biomedical Research and Innovation Institute of Cádiz (INiBICA), Biomedical Research and Innovation Institute of Cádiz (INiBICA), Cadiz, Spain; 3 PROFITH ?PROmoting FITness and Health through physical activity? research group, Department of Physical Education and Sports, Faculty of Sport Sciences, University of Granada, Granada, Spain; 4 Department of Physical Education, Sports and Human Movement, Autonomous University of Madrid, Madrid, Spain; 5 Institute for Physical Activity and Nutrition (IPAN), School of Exercise and Nutrition Science, Deakin University, Geelong, Australia; 6 Faculty of Sport Sciences, University of Extremadura, Caceres, Spain

**Keywords:** prenatal, fitness, birth, anemia, diabetes, children

## Abstract

**Background:**

Physical fitness outcomes are considered major health biomarkers to assess and monitor exercise-based interventions across the lifespan. Recent studies provide evidence that many adult and childhood chronic diseases should have their origins in gestational or fetal life. To date, a few pioneering studies have showed associations between prenatal predictors and selected physical fitness tests (strength and cardiorespiratory). Nevertheless, there is a lack of knowledge about the influence of prenatal factors on childhood performance on a comprehensive fitness test battery including speed and coordination. The innovative purpose of the current study is to analyse the relative weight of prenatal predictors on schoolchildren's physical fitness outcomes.

**Methods:**

We obtain data from1188 children (571 girls) aged 6-11 years and 1020 adolescents (495 girls) aged 12-17 years. Prenatal predictors (gestational anemia, gestational diabetes and length of gestation) were self-reported from offspring's mothers. The ALPHA fitness test battery for youth was used to assess offsprinǵs physical fitness (muscular strength, motor fitness and cardiorespiratory fitness). Regression analysis were performed to predict the different physical fitness outcomes.

**Results:**

The main findings of the present study indicate that the presence of gestational anemia significantly predicted lower scores of lower-body explosive muscular strength (standing long jump) and motor fitness (4x10-m shuttle run) and predicted moderately lower scores of upper-body isometric muscular strength (handgrip strength test). (p>.005; p>.008; p>.075 respectively). Moreover, gestational anemia better predicted lower scores of muscular strength and motor fitness in children than in adolescents (standing long jump, handgrip strength test, 4x10-m shuttle run) (p>.001; p>.051; p > 0.18, respectively). While gestational age and length of gestation (>34- ?42 weeks) predict better cardiorespiratory fitness (20 m shuttle-run test) (p>.023; p>.023 respectively) and motor fitness (4x10 m shuttle; moderately for length of gestation). (p>.020; p > 0.55 respectively).

**Conclusion:**

This evidence suggests that preventive strategies by health-care institutions, policy makers and technicians must be two-fold: a) to effectively reduce gestational anemia in order to prevent offsprinǵs predisposition to low levels of physical fitness, and b) to intervene with toddlers and children at risk to provide tailored physical activity programs and regular physical fitness evaluation.

